# TIM-3 is a potential prognostic marker for patients with solid tumors: A systematic review and meta-analysis

**DOI:** 10.18632/oncotarget.15954

**Published:** 2017-03-07

**Authors:** Yang Zhang, Pengcheng Cai, Tao Liang, Lin Wang, Lihua Hu

**Affiliations:** ^1^ Department of Clinical Laboratory, Union Hospital, Tongji Medical College, Huazhong University of Science and Technology, Wuhan 430022, China; ^2^ Research Center for Tissue Engineering and Regenerative Medicine, Union Hospital, Tongji Medical College, Huazhong University of Science and Technology, Wuhan 430022, China

**Keywords:** TIM-3, meta-analysis, solid tumor, prognostic marker, overall survival

## Abstract

Accumulated studies have demonstrated the important role of T cell immunoglobulin- and mucin-domain-containing molecule-3 (TIM-3) in various solid tumors and indicated its correlation with patients’ survival. To further verify the prognostic significance of TIM-3 in cancer patients and its correlation with tumor, we performed this meta-analysis including seven studies searched from PubMed, Web of Science, and Embase till July 2016. A total of 869 patients were used to analyze the association between TIM-3 expression and patients’ overall survival (OS). The pooled results showed that higher expression of TIM-3 was significantly correlated to shorter OS (7 studies, HR=1.89; 95% CI: 1.38-2.57; P< 0.001). In addition, higher TIM-3 expression was associated with advanced tumor stage (3 studies, III/IV vs. I/II, RR=2.02; 95% CI: 1.45–2.81; P< 0.001). In conclusion, our study highlights the role of TIM-3 as a potential prognostic marker and a promising therapeutic target in solid tumors.

## INTRODUCTION

T cell immunoglobulin- and mucin-domain-containing molecule-3 (TIM-3) belongs to a family of receptors involved in immune-checkpoint functions. It is universally reported to play a crucial role in mediating T cell exhaustion both in viral infections and tumors [1–4]. The expression of TIM-3 was initially identified on CD4 IFN-γ producing cells and in cytotoxic CD8 lymphocytes in mice and humans [5]. Most recent studies have demonstrated that TIM-3 expression on CD4^+^ and CD8^+^ cells is closely related to T cell exhaustion not only in human immunodeficiency virus (HIV) and hepatitis C virus (HCV), but also in cancer patients [6, 7].

A growing number of studies have shown that TIM-3 expression is elevated in a series of solid tumors such as lung cancer [8], gastric cancer [9], colon cancer [10], hepatocellular carcinoma [11], renal cell carcinoma [12], bladder urothelial carcinoma [13], and cervical cancer [14]. Furthermore, the increased TIM-3 level is correlated with poor survival in these tumor patients. In addition, more in-depth research illustrated that the soluble form of TIM-3 reduced the antigen-specific T cell response and downregulated the anti-tumor activity *in vivo* [15]. Another study revealed that blockade of TIM-3 could reverse the impaired phenotype of NK cells in patients with metastatic melanoma [16], which highlights the potential possibility of TIM-3 targeted therapy. Nevertheless, despite all the promising data proved in preclinical models, the role of TIM-3 hasn't been evaluated in clinical trials yet, maybe due to the insufficient evidence of TIM-3′s role in clinical cancer patients.

Therefore, we conducted this meta-analysis, which combined all the published evidence to evaluate the prognostic implication of TIM-3 in patients with solid tumors, thereby to promote the process of genetic diagnosis for cancer and identify novel strategies targeting TIM-3.

## RESULTS

### Studies selection

**Figure 1:**
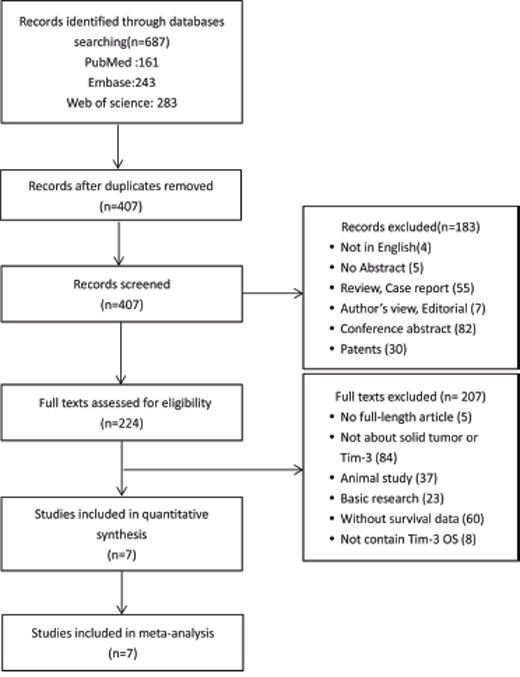
Flow diagram of study selection TIM-3: T cell immunoglobulin- and mucin-domain-containing molecule-3; OS: overall survival.

A total of 687 studies were identified by electronic search and 7 studies were included finally. The details of the study screening process were presented in Figure 1.

### Study characteristics and quality assessment

All seven studies used immunohistochemistry techniques to assess the expression level of TIM-3. A total number of 869 participants were involved in this review. The patients were Asians diagnosed with various cancers involving: bladder urothelial carcinoma (BUC), non–small cell lung cancer (NSCLC), gastric cancer, ovarian cancer, cervical cancer, hepatitis B virus-associated hepatocellular carcinoma and clear cell renal cell carcinoma. The characteristics of the included studies were described in Table 1.

**Table 1:** Characteristics of studies included in the meta-analysis

Author	Year	Country	Cancer type	NO. of patients	Age, median (range)	Male/female	Cancer stage or grade	Percentage of high Tim-3 Cutoff value	Follow-up months	HR and 95%CI
Xuewei Zhuang	2012	Chinese	NSCLC	30	60(37-75)	23/7	TNM:I-IV	15/30(50%), >25% of cells	34(1-78)	NA^a^
Meng Yang	2015	Chinese	Bladder Urothelial Carcinoma	100	65(30-81)	68/32	Grade1-3	50/100(50%), H-score≥100	44(3-60)	NA
Encheng Zhou	2015	Chinese	Colon Cancer	201	65(26-90)	116/85	TNM:I-IV	118/201(58.7%), HSCORE≥200	61(2-103)	NA
Jing Jiang	2013	Chinese	Gastric Cancer	305	64(32-87)	231/74	TNM:I-IV	183/305(60%), HSCORE>0	40(3-135)	NA
Yang Cao	2013	Chinese	Cervical Cancer	43	39(27-67)	0/43	TNM:I-IV	28/43(65.1%), IRS scores2 and 3	45.2(5-60)	NA
Hang Li	2012	Chinese	Hepatocellular Carcinoma	99	51(38-72)	91/8	TNM:I-IV	57/99, NA	36	NA
Yoshihiro Komohara	2015	Japanese	Clear Cell Renal Cell Carcinoma	91	NA	59/32	Grade1-4	63/92(68.5%), score 1,2	120	HR:3.7 CI(0.7–68) P=0.12

^a^ NA: not available.

The results from the quality of included studies reveals that the selection bias exists in each included study, as each study included one type of cancer, which cannot represent the whole population of solid tumor. Three studies [9, 11, 12] did not state whether the assessment of TIM-3 positive or high expression was evaluated by blinded pathologists, therefore the ascertainment of exposure is of some concern. For the comparability, two studies use reported unadjusted data [12, 13] which may induce confounding bias. Other studies [8, 10, 14] used adjusted data (Cox multivariate analysis) to assess the prognostic value of TIM-3 for overall survival. As the prognostic outcomes are survival, so the outcome assessment is objective and the follow up is long enough for outcomes to occur (Table 2).

**Table 2:** The Newcastle-Ottawa Scale (NOS) quality assessment of the enrolled studies

Study ID	SELECTION			COMPARABILITY			OUTCOME		Total
	Representativeness of the exposed cohort	Selection of the non-exposed cohort	Ascertainment of exposure	Demonstration that outcome of interest was not present at start of study	Comparability of cohorts on the basis of the design or analysis (study adjusts for age*, sex*)	Assessment of outcome	Was follow-up long enough for outcomes to occur	Adequacy of follow up of cohorts	
Xuewei Zhuang 2012 [8]	-	-	*	*	**	*	*	*	7
Meng Yang 2015 [13]	-	-	-	*	*	*	*	*	5
Encheng Zhou 2015 [10]	-	-	*	*	**	*	*	*	7
Jing Jiang 2013 [9]	-	-	*	*	**	*	*	*	7
Yang Cao 2013 [14]	-	-	-	*	**	*	*	*	6
Hang Li 2012 [11]	-	-	-	*	**	*	*	*	6
Yoshihiro Komohara 2015 [12]	-	-	*	*	-	*	*	*	5

### Meta-analysis

#### The prognostic value of TIM-3 in solid tumor patients’ overall survival

**Figure 2:**
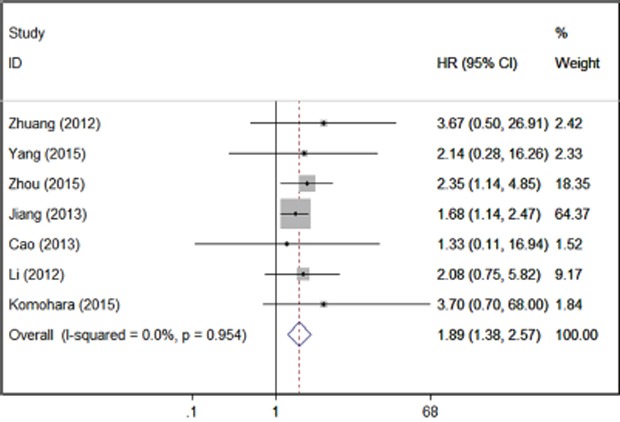
Forrest plots of studies evaluating TIM-3 expression level and patients’ overall survival

Seven studies were included in the meta-analysis of tumor patients’ OS. A fixed effect model was used to calculate the pooled HR and 95% CI, as the heterogeneity test reported a P value of 0.954 and I^2^ value of 0.0%. The results showed that patients with higher expression of TIM-3 had significant shorter overall survival (7 studies, n=869, HR=1.89; 95% CI: 1.38-2.57; P< 0.001). Combing all the tumor types, the results indicated an association between TIM-3 and patients’ OS (Figure 2).

#### Association of TIM-3 with clinicopathological parameters

**Figure 3:**
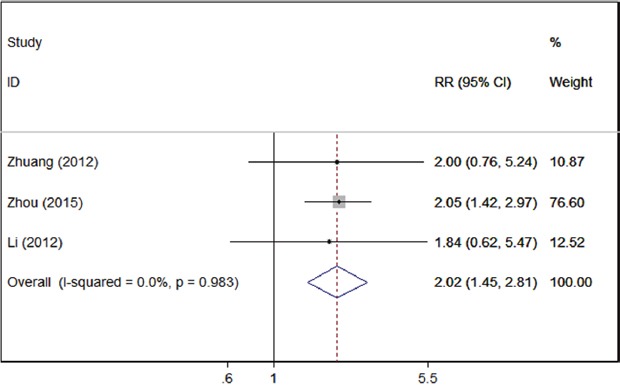
Forrest plots of studies evaluating TIM-3 expression and TNM stage

In the comprehensive analysis for the role of Tim-3 expression in solid tumor as a biomarker, we also investigated the association of high TIM-3 expression and clinicopathological characteristics. As shown by Table 3, TIM-3 expression had no obvious association with patients’ age, sex or T stage; however, high TIM-3 expression was significantly associated with advanced TNM stage (3 studies, n=331, III/IV vs. I/II, RR=2.02; 95% CI: 1.45–2.81; p < 0.001) (Figure 3).

**Table 3:** Correlation of TIM-3 expression and clinical features

Variables	Cancer type	Studies	Pooled RR	95% CI	Model	Heterogeneity I^2^ (%)	P Value
Age	Overall	3	0.889	0.678-2.605	fixed	0.0	0.264
	Non–small cell lung cancers	1	0.971	0.747-1.263			
	Bladder urothelial carcinoma	1	0.820	0.579-1.161			
	Gastric cancer	1	0.750	0.344-1.636			
Sex	Overall	4	0.976	0.757-1.258	fixed	0.0	0.850
	Non–small cell lung cancers	1	1.333	0.358-4.965			
	Bladder urothelial carcinoma	1	0.778	0.436-1.386			
	Colon cancer	1	1.055	0.758-1.469			
	Clear cell renal cell carcinomas	1	0.928	0.511-1.686			
T stage	Overall	4	2.464	0.104-58.422	random	99.4	0.577
	Non–small cell lung cancers	1	1.571	0.844-2.924			
	Bladder urothelial carcinoma	1	9.750	3.765-25.247			
	Colon cancer	1	0.976	0.942-1.012			
	Clear cell renal cell carcinomas	1	2.531	1.306-4.906			
TNM stage	Overall	3	1.654	0.641-4.270	fixed	0.0	< 0.001
	Non–small cell lung cancers	1	2.000	0.763-5.242			
	Colon cancer	1	2.052	1.419-2.967			
	Hepatocellular carcinoma	1	1.842	0.620-5.473			

#### Publication bias

**Figure 4:**
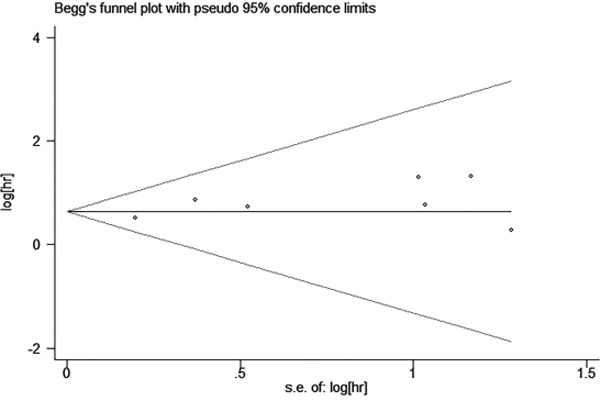
Begg's funnel plot estimating the publication bias of the included literatures

Begg's funnel plot and Egger's test were used to estimate the publication bias of the included literatures. The shapes of the funnel plots for the OS showed no evidence of obvious asymmetry (Figure 4), and Egger's test revealed non-significant value (P =0.134).

#### Sensitivity analysis

**Figure 5:**
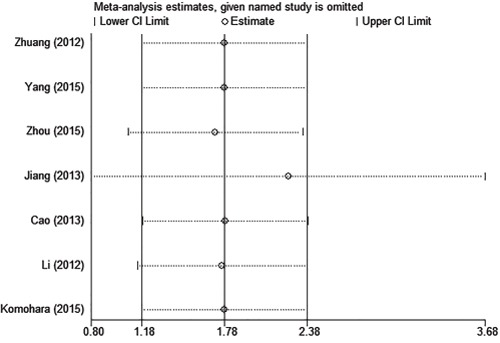
Sensitivity analysis of this meta-analysis

Sensitivity analysis was carried out to assess the influence of individual study on the synthetic results of OS. The results showed that the pooled HR was not significantly influenced after omitting any single study for the effect of TIM-3 expression on OS (Figure 5).

## DISCUSSION

As far as we know, our current meta-analysis may be the only evidence evaluating the association between TIM-3 expression and patients’ overall survival in solid tumor. We systematically evaluated survival data for 869 patients and demonstrated a positive relationship between TIM-3 high expression and poor prognosis in cancer patients. Besides, we also investigated the association between TIM-3 expression and clinicopathological characteristics. Results support that TIM-3 high expression is significantly correlated with advanced tumor stage, which indicates that TIM-3 probably participates in tumor progression and finally affects tumor prognosis.

It has been demonstrated that T cell exhaustion could promote tumorigenesis and tumor progression in various cancers [17], and TIM-3 is one of the most important molecules that mediate T cell exhaustion [18]. Accumulating evidences have revealed that TIM-3 contributed to tumor-initiating and tumor-promoting activities [19, 20]. Huang et al found that TIM-3 could facilitate the onset, growth and dissemination of lymphoma by suppressing activation of CD4^+^ T cell through the interleukin-6-STAT3 pathway [21]. Our lab recently identified that TIM-3^+^ CD8^+^ T cell have impaired ability of IFN-γ production which inhibited the cytotoxic activity of functional T cells [22]. Another study conducted by Yu-Hwa Huang found that administration of anti-mTIM-3 to mouse models with colon cancer delayed tumor growth. Moreover, TIM-3 blockade was associated with enhanced IFN-γ production, which indicated restoration of T cell function [23]. All these studies illustrated that TIM-3 may promote cancer progression through IL-6/STAT3 pathway or inhibiting IFN-γ production of effective T cells against tumor cells, exhibiting the function as a tumor-promoting role.

In the case of immunosuppressive prognostic markers, there are others like CTLA-4 (cytotoxic T-lymphocyte antigen-4), LAG3 (Lymphocyte-activation gene 3) and PD-1(programmed death-1) have been identified as well as TIM-3 [24]. Furthermore, some of them have been applicated in the immunotherapy for malignant tumors [25, 26]. Unfortunately, there are no optimal targets so far, although the PD-1 blockage has shown some encouraging effects and has been implicated in some clinical trials, however, the benefits and improved prognosis for cancer patients remain unsatisfactory [27]. Our study implies that TIM-3 may be a prognostic marker of patients’ survival with solid tumors, and high expression of TIM-3 may correlate with advanced tumor stage. These findings reveal that TIM-3 may be an emerging target in cancer therapy. Interestingly, there are several preclinical studies which show that combined targeting of TIM-3 and PD-1 pathways is more effective in controlling tumor growth than targeting either pathway alone [3]. It is promising to see the responses of combined immunotherapies.

In the meanwhile, there are some limitations for this paper as well. First of all, all the enrolled participants were Asians and could not represent the whole population; secondly, some of the studies are in small scale, which include patients less than 40; thirdly, the quality of the included studies is with selection bias, as each study included only one type of cancer, which also exhibiting the urgency for more studies with larger sample size regarding this.

In conclusion, TIM-3 seems not merely a valuable prognostic marker but also a promising therapeutic target for solid tumors. Due to the sparse data, more studies regarding TIM-3 are still required.

## MATERIALS AND METHODS

### Literature search

We searched for papers published in PubMed, Web of Science and Embase on July 4, 2016. The following keywords were used to perform the search: “HAVCR2/T-cell immunoglobulin and mucin domain containing 3”, “TIM-3”, “T-cell Ig and mucin domain 3”, “hepatitis A virus cellular receptor 2”, “cancer”, “tumor”, “carcinoma”, “neoplasm”, “prognosis”, “survival”, “mortality” and “death”.

Three review authors screened the studies with the following inclusion criteria: i) studies investigating the association of TIM-3 with prognostic outcomes in solid tumor; ii) The study designs were cross-sectional study, cohort study or case-control study; iii) The interested prognostic outcomes include overall survival and tumor progression (tumor stage and histological grade); iv) The study was published in English [28]. We will exclude animal studies and studies with the length of follow-up less than 3 years. Studies that are not available in full text will also be excluded. Any disagreement between the three authors will be resolved by consensus.

### Data collection and quality assessment

Three investigators independently extracted the data from eligible studies using a predefined form. The collected data included the name of first author, publication year, patients’ country of origin, tumor type, number of patients, age, sex, cancer stage or grade, detection method for TIM-3 expression, percentage exhibiting high TIM-3 expression and the corresponding cutoff value, median follow-up months, outcome, HR and 95%CI of high TIM-3 expression group versus low.

For studies that presented only Kaplan-Meier curves, Engauge Digitizer (version 4.1) was used to extract the survival data. The estimated HRs and 95% CIs were calculated by Tierney's method [28]. Three review authors independently assessed the quality of included studies by using Newcastle-Ottawa Quality Assessment Scale (NOS). Three domains were evaluated including selection of participants, comparability, and ascertainment of outcome.

### Statistical analysis

Stata version 14.0 (Stata Corporation, College Station, TX, USA) was used to carry out the statistical analysis. Pooled HRs and 95% CIs for OS were used to assess the association between TIM-3 expression and overall survival. In addition, RRs and their 95% CIs were used to assess the correlation between TIM-3 expression and the clinicopathological features of each solid tumor. Heterogeneity was assessed by the I^2^ value derived from Q test [29] coupled with a P value from Chi square test. We considered a P value of less than 0.10 and I^2^ >50% as significant heterogeneity. Fixed effect model was initially applied to combine the estimates of effect, while a substantial heterogeneity between studies was observed and the source of heterogeneity was identified, otherwise a random effects model was used to combine the data [30].

### Publication bias and sensitivity analysis

Publication bias was tested using Begg's funnel plot and the Egger's test [31]. If the funnel plot is asymmetric and the Egger's test reported a P value of less than 0.05, publication bias is deemed to probably exist.

Meanwhile, we performed the sensitivity analysis for overall survival by omitting each study to assess the influence of individual study on the whole meta-analysis.
